# Increased Set1 binding at the promoter induces aberrant epigenetic alterations and up-regulates cyclic adenosine 5'-monophosphate response element modulator alpha in systemic lupus erythematosus

**DOI:** 10.1186/s13148-016-0294-2

**Published:** 2016-11-24

**Authors:** Qing Zhang, Shu Ding, Huilin Zhang, Hai Long, Haijing Wu, Ming Zhao, Vera Chan, Chak-Sing Lau, Qianjin Lu

**Affiliations:** 1 Department of Dermatology, Second Xiangya Hospital, Central South University, Changsha, Hunan 410011 China; 2 Department of Dermatology, Third Xiangya Hospital, Central South University, Changsha, Hunan 410011 China; 3 Emergency Department, Second Xiangya Hospital, Central South University, Changsha, Hunan 410011 China; 4 Division of Rheumatology and Clinical Immunology, Department of Medicine, The University of Hong Kong, Hong Kong, China

**Keywords:** Systemic lupus erythematosus, CREMα, H3K4me3, Set1, DNA methylation, DNMT3a

## Abstract

**Background:**

Up-regulated cyclic adenosine 5'-monophosphate response element modulator α (CREMα) which can inhibit IL-2 and induce IL-17A in T cells plays a critical role in the pathogenesis of systemic lupus erythematosus (SLE). This research aimed to investigate the mechanisms regulating CREMα expression in SLE.

**Results:**

From the chromatin immunoprecipitation (ChIP) microarray data, we found a sharply increased H3 lysine 4 trimethylation (H3K4me3) amount at the CREMα promoter in SLE CD4+ T cells compared to controls. Then, by ChIP and real-time PCR, we confirmed this result. Moreover, H3K4me3 amount at the promoter was positively correlated with CREMα mRNA level in SLE CD4+ T cells. In addition, a striking increase was observed in SET domain containing 1 (Set1) enrichment, but no marked change in mixed-lineage leukemia 1 (MLL1) enrichment at the CREMα promoter in SLE CD4+ T cells. We also proved Set1 enrichment was positively correlated with both H3K4me3 amount at the CREMα promoter and CREMα mRNA level in SLE CD4+ T cells. Knocking down Set1 with siRNA in SLE CD4+ T cells decreased Set1 and H3K4me3 enrichments, and elevated the levels of DNMT3a and DNA methylation, while the amounts of H3 acetylation (H3ac) and H4 acetylation (H4ac) didn’t alter greatly at the CREMα promoter. All these changes inhibited the expression of CREMα, then augmented IL-2 and down-modulated IL-17A productions. Subsequently, we observed that DNA methyltransferase (DNMT) 3a enrichment at the CREMα promoter was down-regulated significantly in SLE CD4+ T cells, and H3K4me3 amount was negatively correlated with both DNA methylation level and DNMT3a enrichment at the CREMα promoter in SLE CD4+ T cells.

**Conclusions:**

In SLE CD4+ T cells, increased Set1 enrichment up-regulates H3K4me3 amount at the CREMα promoter, which antagonizes DNMT3a and suppresses DNA methylation within this region. All these factors induce CREMα overexpression, consequently result in IL-2 under-expression and IL-17A overproduction, and contribute to SLE at last. Our findings provide a novel approach in SLE treatment.

**Electronic supplementary material:**

The online version of this article (doi:10.1186/s13148-016-0294-2) contains supplementary material, which is available to authorized users.

## Background

Systemic lupus erythematosus (SLE) is a chronic autoimmune disease which multiple pathogenic mechanisms are involved in [1, 2]. Recently, accumulating studies have documented that epigenetic alterations in certain genes of T cells play critical roles in the pathogenesis of SLE [3, 4]. Epigenetics refers to heritable changes in gene expression without changes in the DNA sequence [5, 6]. The epigenetic mechanisms include mainly DNA methylation, histone modifications, noncoding RNA regulation, and chromatin modifications [5, 7]. It has been proved that DNA methylation is hallmark of gene silencing [8], while H3 lysine 4 trimethylation (H3K4me3), H3 acetylation (H3ac), and H4 acetylation (H4ac) are all correlated with transcriptional activation [9–11]. As one of the most familiar histone modifications, H3K4me3 is always a focus of epigenetics. It accumulates predominantly at the promoters and early transcribed regions of active genes, and is involved in transcription initiation, elongation and RNA processing by interacting with RNA polymerase II [12, 13]. It also can recruit and/or stabilize chromatin-remodeling enzymes and transcriptional cofactors [14, 15]. Interestingly, H3K4me3 is able to inhibit DNA methylation by antagonizing DNA methyltransferase (DNMT) 3a [16], and augment histone acetylation by interacting with histone acetyltransferases (HATs) [17]. As we all know, histone methyltransferases (HMTs) SET domain containing 1 (Set1) and mixed-lineage leukemia 1 (MLL1) can both catalyze trimethylation of H3K4 [18, 19].

Set1 and MLL1 are both large proteins containing one C-terminal SET domain that is associated with an intrinsic histone lysine-specific methyltransferase activity [20–22]. They are present, respectively, as the catalytic subunit and central element of multi-protein H3K4 methyltransferase complexes named complex of proteins associated with Set1 (COMPASS) and COMPASS-like [23–25]. Besides the catalytic Set1/MLL1 subunit, COMPASS/COMPASS-like contains several other proteins. Set1/MLL1 protein alone possesses very weak HMT activity, and their full activities require the context of the whole complexes [26, 27].

T cells from SLE patients and murine models produce less IL-2 compared to normal controls, and lower IL-2 level in SLE patients with higher SLE Disease Activity Index (SLEDAI) [28, 29]. Decreased IL-2 expression results in impaired generation of cytotoxic responses, reduced number and function of T regulatory cells (Tregs), and defective activation-induced cell death (AICD). In SLE patients, various cytotoxic responses have been reported ineffective and may account for the increased susceptibility to infection. The inhibited Tregs are unable to prevent autoimmunity, and the deficiency in AICD may lead to extended survival of autoreactive T cells, thereby B cells overactivate, in the end, resulting in overproduction of autoantibodies and the development of SLE [30–32].

Contrary to IL-2, T cells from patients with SLE and SLE murine models produce higher amounts of IL-17A, and IL-17A level is positively correlated with disease activity of SLE and titer of anti-dsDNA. Concordantly, inhibition of IL-17A can decrease the manifestations of lupus [33–36]. IL-17A is able to interact with various chemokines and cytokines, consequently triggers profound proinflammatory responses. It also stimulates B cells to proliferate and product more antibodies (including total IgG, anti-DNA and anti-histone antibodies) [28, 37, 38]. All these contribute to the onset of SLE.

Among the factors that regulate IL-2 and IL-17A, the cyclic adenosine 5'-monophosphate (cAMP) response element modulator α (CREMα) plays crucial roles in SLE. It has been reported that CREMα is increased in T cells from SLE patients, and the CREMα promoter activity is positively correlated with SLE disease activity [29]. The overexpression of CREMα can suppress TCR/CD3ζ chain transcription, which is able to terminate the T cell response. It also represses the transcription factor c-fos, the antigen-presenting cell molecule CD86, and Notch signaling receptor Notch-1 to participate in the pathogenesis of SLE [35, 39–41]. And, the most important mechanism is that overexpressing CREMα can repress IL-2, yet increased IL-17A [32, 42]. However, which factors and mechanisms contribute to increased CREMα in SLE T cells remain unclear.

Through methylated CpG-DNA immunoprecipitation (MeDIP), Hedrich CM et al. found that DNA methylation level at the CREMα promoter in SLE CD4^+^ T cells is lower than healthy controls; moreover, CREMα promoter methylation is reduced in SLE patients who were in active stage compared to the patients in remission [29]. By chromatin immunoprecipitation (ChIP) microarray, we found that H3K4me3 enrichment at the CREMα promoter was significantly higher in SLE CD4^+^ T cells than in healthy controls. We then confirmed this result by ChIP and real-time PCR. In addition, a marked increase in Set1 binding was observed, but no striking change in MLL1 binding at the CREMα promoter in CD4^+^ T cells of patients with SLE. Knocking down Set1 with siRNA in SLE CD4^+^ T cells resulted in reduced both Set1 binding and H3K4me3 enrichment at the CREMα promoter, thus suppressing the expression of CREMα, and increasing the amount of IL-2, simultaneously decreasing the production of IL-17A. We also found the levels of both DNA methylation and DNMT3a were elevated, while the concentrations of H3ac and H4ac did not change greatly within the CREMα promoter in SLE CD4^+^ T cells whose Set1 was knocked down. According to this clue, we further verified that DNMT3a was decreased within the CREMα promoter in SLE CD4^+^ T cells, and H3K4me3 enrichment was negatively correlated with both DNA methylation level and DNMT3a binding at the promoter. Taken together, these results provide novel insights into the epigenetic mechanisms that cause SLE.

## Methods

### Subjects

Twenty SLE patients (age 27.10 ± 6.52 years) were recruited from the out-patient clinics and in-patient wards of the Second Xiangya Hospital, Central South University, China. All patients fulfilled at least four of the SLE classification criteria of the American College of Rheumatology (ACR) [43]. Relevant clinical information of the SLE patients is listed in Table 1. Twenty healthy donors (age: 28.20 ± 5.21 years) were recruited from medic staff and graduate students at the Second Xiangya Hospital. All patients and controls were age- and sex-matched, and written informed consent was obtained from every participant. This study was approved by the Human Ethics Committee of the Central South University Second Xiangya Hospital and was conducted in accordance with the Declaration of Helsinki.

**Table 1:** Profiles of patients with SLE

Patient	Gender	Age (years)	SLEDAI	Medications
1	Female	23	8	Pred 30 mg/d
2	Female	20	6	Pred 20 mg/d
3	Male	38	7	Pred 20 mg/d
4	Female	21	3	None
5	Female	26	12	None
6	Female	28	12	Pred 40 mg/d
7	Female	35	4	HCQ 0.2 g/d
8	Female	19	2	None
9	Female	33	3	Pred 5 mg/d
10	Female	27	2	None
11	Female	32	15	Pred 40 mg/d, TG^c^ 30 mg/d
12	Female	22	4	HCQ 0.2 g/d
13	Female	20	3	Pred 5 mg/d
14	Female	22	10	Pred 30 mg/d, TG 30 mg/d
15	Female	25	0	None
16	Male	40	10	Pred 40 mg/d, HCQ 0.2 g/d
17	Female	30	16	Pred 50 mg/d, TG 30 mg/d
18	Female	26	2	HCQ 0.2 g/d
19	Female	20	8	None
20	Female	35	12	Pred 35 mg/d, HCQ 0.2 g/d

*SLEDAI* systemic lupus erythematosus, *Pred* prednisone, *HCQ* hydroxychloroquine, *TG* tripterygium glycoside

### Cell isolation

Peripheral blood mononuclear cells (PBMCs) were isolated by Ficoll-Hypaque density gradient centrifugation (GE Healthcare), and CD4^+^ T cells were subsequently isolated by positive selection using magnetic beads (Miltenyi), according to the manufacturer’s instruction. The purity of enriched CD4^+^ T cells was generally higher than 95%, as checked by flow cytometry.

### ChIP microarray

CD4^+^ T cells from five SLE patients (relevant clinical information is listed in Additional file 1: Table S1) and five age- and sex-matched healthy controls were fixed with 1% formaldehyde for 10 min, then they were lysed by lysis buffer. Lysates from SLE patients and healthy controls were pooled respectively, and were sent to Capitalbio (Beijing, China). ChIP microarray quality control, labeling, hybridization, scanning, and statistical analyze were carried out by Capitalbio. Anti-H3K4me3 antibody-precipitated DNA and total DNA (input) were labeled with Cy5 (red) and Cy3 (green), respectively. Samples were then cohybridized onto the microarray panels, subsequently Cy3/Cy5 ratio images of the microarray were generated. In these images, diversified color intensities represented relative H3K4me3 enrichments at various gene promoters. Compared to control CD4^+^ T cells, at least twofold increase or decrease in H3K4me3 enrichments in SLE CD4^+^ T cells were considered significant.

### ChIP and real-time PCR

ChIP assay was performed using a ChIP kit (Millipore), according to the instruction provided by the manufacturer. Briefly, CD4^+^ T cells were fixed with 1% formaldehyde for 10 min, then lysed with lysis buffer. Cell lysates were sonicated to shear the DNA, subsequently the sonicated extracts were clarified by centrifugation. After preclearing by protein G-agarose beads, antibodies were added and incubated with the extracts at 4 °C overnight on a rotator. The next day, protein G-agarose beads were added and rotated for 1 h at 4 °C to pull down immunoprecipitated complexes. The complexes were washed and subsequently eluted with elution buffer. After reversing cross links between DNA and protein by heating at 65 °C for 4 h, the DNA was purified and subjected to real-time PCR analysis, and the input DNA was used as endogenous control. All experiments were performed three times. The primers for CREMα promoter were: forward 5′-TGGGGAGATAGAGGTTGCAG-3′ and reverse 5′-CGCCAGAAATCCAATGACTT-3′. The anti-H3K4me3 antibody, anti-H3ac antibody, and anti-H4ac antibody were purchased from Millipore, and the anti-Set1 antibody, anti-MLL1 antibody, and anti-DNMT3a antibody were from Abcam.

### RNA extraction and real-time RT-PCR

Total RNA was isolated from CD4^+^ T cells using TRIzol Reagent (Invitrogen) according to the protocol provided by the manufacturer, and stored at −80 °C. Real-time RT-PCR was performed with a Rotor-Gene3000 thermocycler (Corbett Research), and mRNA level was quantified by a SYBR PrimeScript RT-PCR kit (Takara). β-actin was amplified simultaneously as an endogenous control. Negative control (using water instead of RNA) was also run for every experiment. All reactions were run in triplicate. Primers used were as follows: for CREMα, forward 5’-GAAACAGTTGAATCCCAGCATGATGGAAGT-3’ and reverse 5’-TGCCCCGTGCTAGTCTGATATATG-3’; for β-actin, forward 5’-CGCGAGAAGATGACCCAGAT-3’ and reverse 5’-GCACTGTGTTGGCGTACAGG-3’.

### Transfection

Control-siRNA and Set1-siRNA were all designed and synthesized at Guangzhou RiboBio in China. SiRNA transfections were performed with a Human T Cell Nucleofector kit and a nucleofector (Amaxa), according to the protocols provided by the manufacturer. The transfected CD4^+^ T cells were then cultured in human T cell culture medium containing 10% fetal bovine serum (FBS). 24 h after transfection, the cells were stimulated with 5.0 μg/ml anti-CD3 and 2.5 μg/ml anti-CD28 antibodies for 48 h, in order to activate CD4^+^ T cells. Whereafter, they were subjected to further analysis.

### Western blotting

CD4^+^ T cells were lysed with whole cell lysis buffer, and proteins were extracted and separated by SDS-polyacrylamide gel electrophoresis, then they were transferred to PVDF membranes (Millipore). The membranes were blocked in TBST buffer containing 5% non-fat milk, and incubated overnight at 4 °C with CREMα antibody (1:500, Abcam), Set1 antibody (1:500, Abcam), or β-actin antibody (1:1000, Santa Cruz). All experiments were repeated three times, and relative expression levels were quantified by Quantity One software (Bio-Rad).

### ELISA

IL-2 and IL-17A productions in the supernatants of stimulated T cells were measured by IL-2 and IL-17A quantification ELISA kits respectively (Yuanxiang), both following the manufacturer’s instructions. Three replicate wells were used for every sample, and all experiments were performed three times. OD values were read at 450 nm for both IL-2 and IL-17A quantification.

### MeDIP and real-time PCR

The methylated CpG-DNA immunoprecipitation assay was performed following the manufacturer’s instruction (Abcam). Briefly, cells were lysed by lysis buffer, and DNA was sheared to fragments of 200–1000 bp by sonication. After centrifuging, the clear supernatants were incubated with antibody for 5-methylcytosine or normal mouse IgG as the negative control. Subsequently, methylated CpG-DNA was released from immunoprecipitated complexes. After purifying, the DNA was subjected to real-time PCR analysis, with input DNA as endogenous control. All experiments were performed in triplicate.

### Statistical analysis

Results were presented as mean ± SD. Values were compared by Student’s *t* test (paired *t* test was used to compare data from different transfections, and two-group *t* test was used to compare others). Correlations were measured by Pearson’s correlation coefficient. *P* values less than 0.05 were considered significant. All results were analyzed with SPSS 16.0 software (SPSS Inc.).

## Results

### Increased H3K4me3 enrichment at the CREMα promoter in SLE CD4^+^ T cells in the results of ChIP microarray

**Fig. 1:**
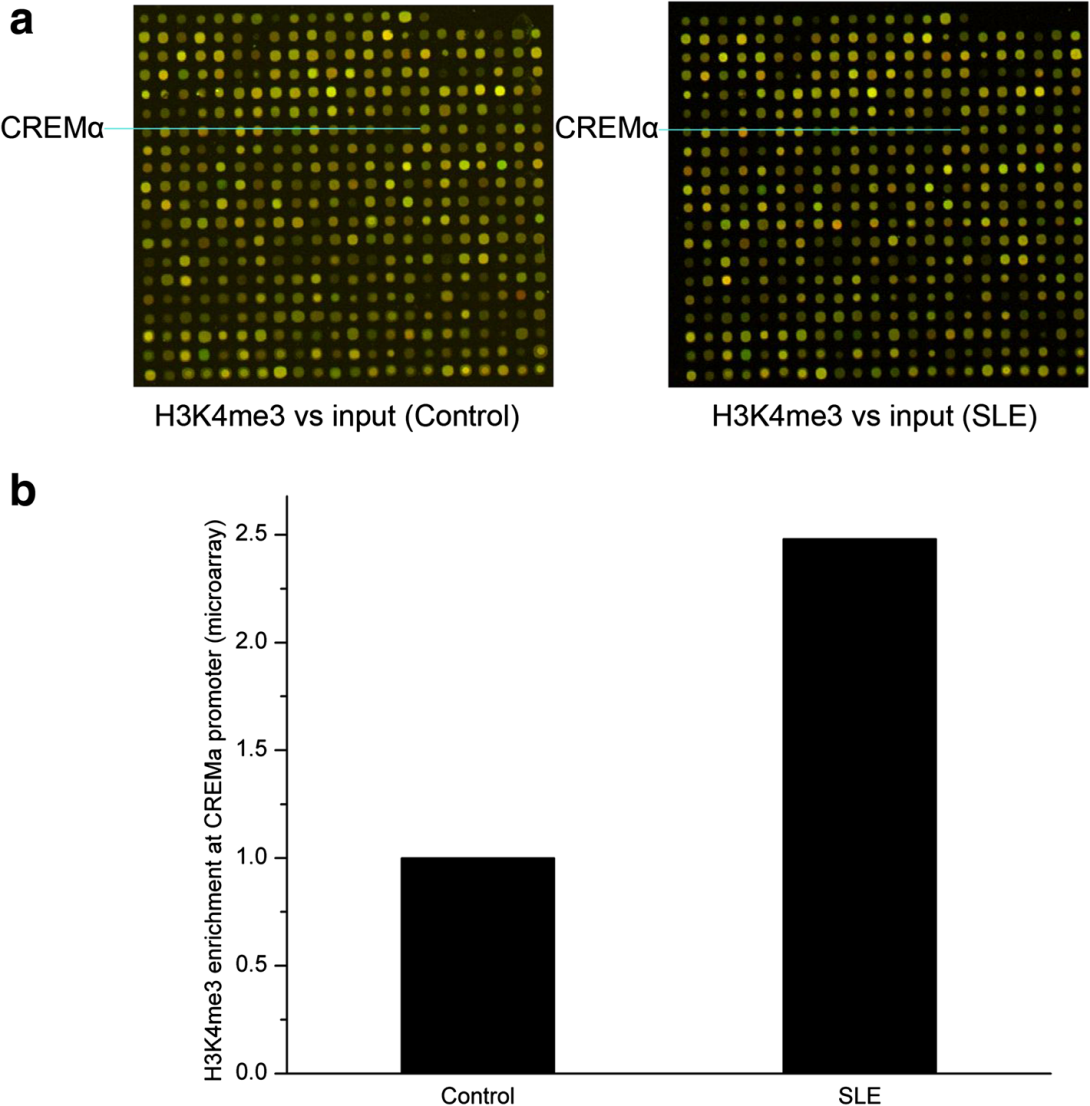
ChIP microarray analysis of H3K4me3 enrichments in SLE and control CD4^+^ T cells. **a** ChIP microarray panels showing relative H3K4me3 enrichments at various gene promoters in CD4^+^ T cell lysates pooled from five healthy controls (*left-hand panel*) and five patients with SLE (*right-hand panel*). Anti-H3K4me3 antibody-precipitated DNA and total DNA (input) were respectively labeled with Cy5 (*red*) and Cy3 (*green*), and samples were subsequently cohybridized onto microarray panels. Each individual *dot* shows the Cy3/Cy5 ratio representing relative H3K4me3 enrichment at a specific gene promoter. The CREMα promoter dot (indicated by a *blue line*) is located in the sixteenth column, seventh row. **b** Relative H3K4me3 enrichment at the CREMα promoter in SLE and control CD4^+^ T cells, quantified from the results shown in (**a**)

We first used ChIP microarray to measure H3K4me3 enrichments at various gene promoters in pooled CD4^+^ T cell lysates from SLE patients and healthy controls. Based on the microarray results, out of the total 20,832 distinct gene promoters screened, 493 showed a greater than twofold difference in H3K4me3 enrichments between the two groups. Among these, H3K4me3 enrichment at the CREMα promoter in SLE CD4^+^ T cells was 2.48 times higher than in control CD4^+^ T cells (Fig. 1a, b).

### Increased H3K4me3 enrichment at the CREMα promoter in SLE CD4^+^ T cells

**Fig. 2:**
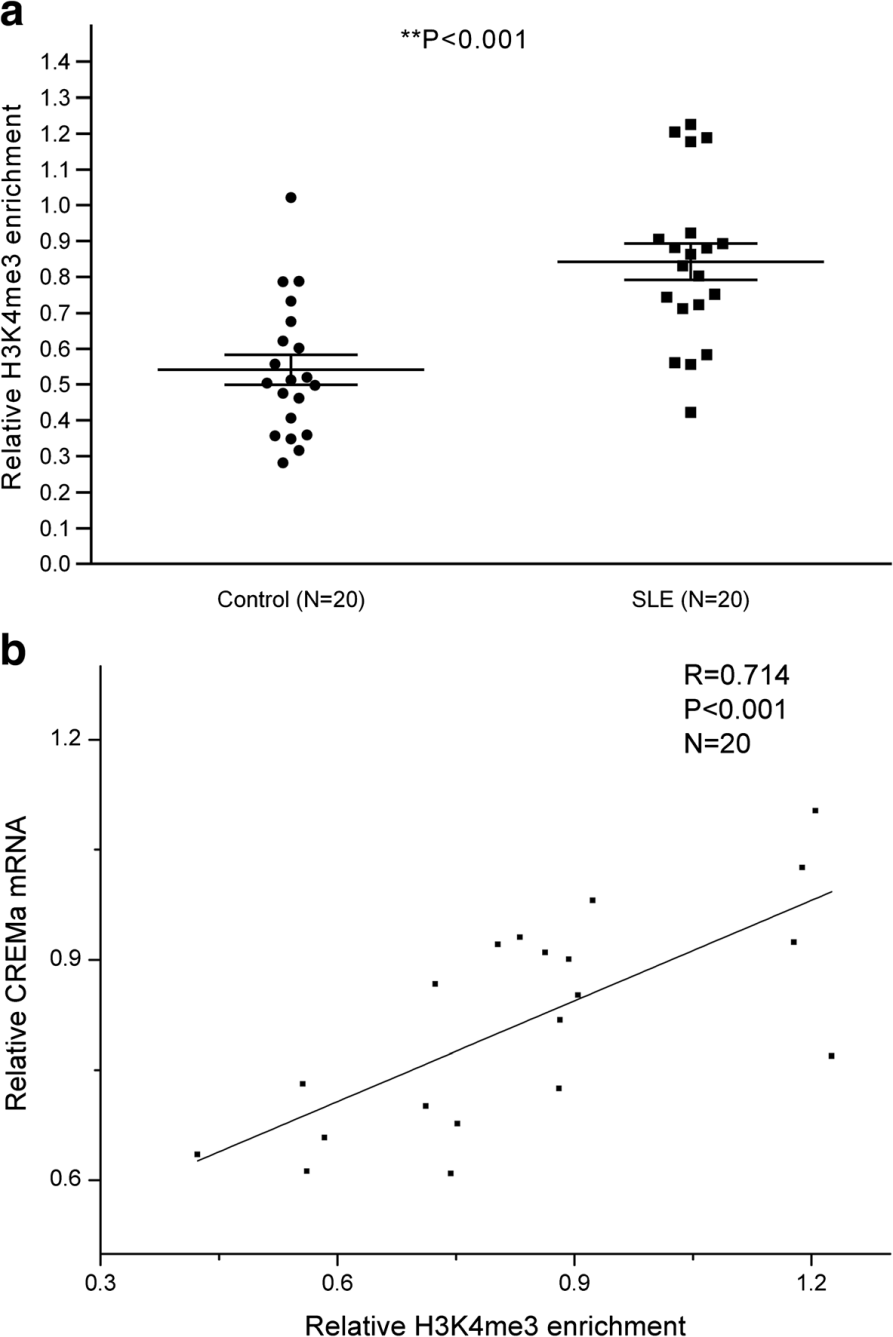
H3K4me3 enrichment at the CREMα promoter in SLE and control CD4^+^ T cells. **a** Relative H3K4me3 enrichment within the CREMα promoter in SLE and healthy CD4^+^ T cells was assessed by ChIP and real-time PCR. Results were normalized to input DNA (total chromatin). **b** Positive correlation between the levels of H3K4me3 and CREMα mRNA in SLE CD4^+^ T cells. All reactions were run in triplicate

In order to verify the finding of ChIP microarray, ChIP and real-time PCR were performed to measure H3K4me3 enrichment at the CREMα promoter in CD4^+^ T cells from 20 SLE patients and 20 healthy controls. Compared to healthy controls, H3K4me3 enrichment at the CREMα promoter was significantly increased in SLE CD4^+^ T cells (Fig. 2a, Additional file 1: Table S2), consistent with our ChIP microarray result. We further carried out real-time RT-PCR to examine CREMα mRNA level in CD4^+^ T cells from SLE patients, and documented that H3K4me3 enrichment at the promoter was positively correlated with CREMα mRNA level in SLE CD4^+^ T cells (Fig. 2b).

### Up-regulated Set1 binding at the CREMα promoter in SLE CD4^+^ T cells

**Fig. 3:**
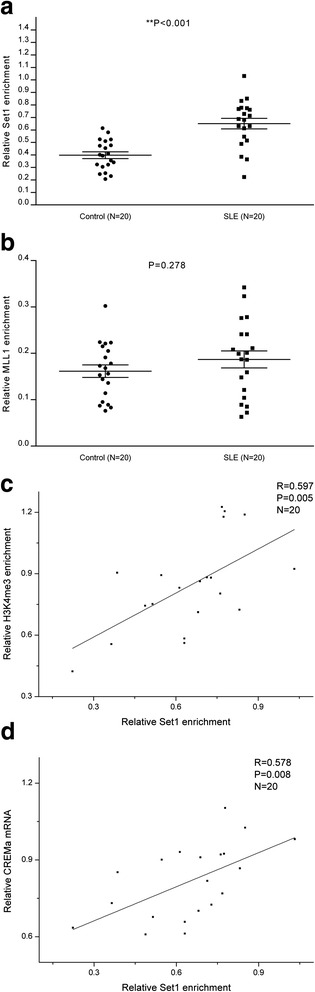
Set1 and MLL1 binding at the CREMα promoter in SLE and control CD4^+^ T cells. **a**, **b** Relative levels of Set1 (**a**) and MLL1 (**b**) binding within the CREMα promoter region in SLE and healthy CD4^+^ T cells were analyzed by ChIP and real-time PCR. Results were normalized to input DNA (total chromatin). **c** Positive correlation between Set1 promoter binding and H3K4me3 level in SLE CD4^+^ T cells. **d** Positive correlation between Set1 promoter binding and CREMα mRNA level in SLE CD4^+^ T cells. All experiments were repeated three times

Overexpression of H3K4me3 at the CREMα promoter in SLE CD4^+^ T cells prompted us to evaluate the status of two H3K4 methyltransferases, Set1 and MLL1. ChIP followed by real-time PCR was carried out to detect the levels of Set1 and MLL1 binding at the CREMα promoter in CD4^+^ T cells from the 20 SLE patients and 20 healthy controls. A marked increase was identified in Set1 binding at the CREMα promoter in SLE CD4^+^ T cells compared with controls (Fig. 3a, Additional file 1: Table S2). However, MLL1 binding at the CREMα promoter did not demonstrate significant difference between SLE and control groups (Fig. 3b, Additional file 1: Table S2). In addition, we confirmed that the level of Set1 binding was positively correlated with both H3K4me3 enrichment at the CREMα promoter (Fig. 3c) and CREMα mRNA level in SLE CD4^+^ T cells (Fig. 3d).

### Down-regulating Set1 in SLE CD4^+^ T cells inhibits CREMα expression

**Fig. 4:**
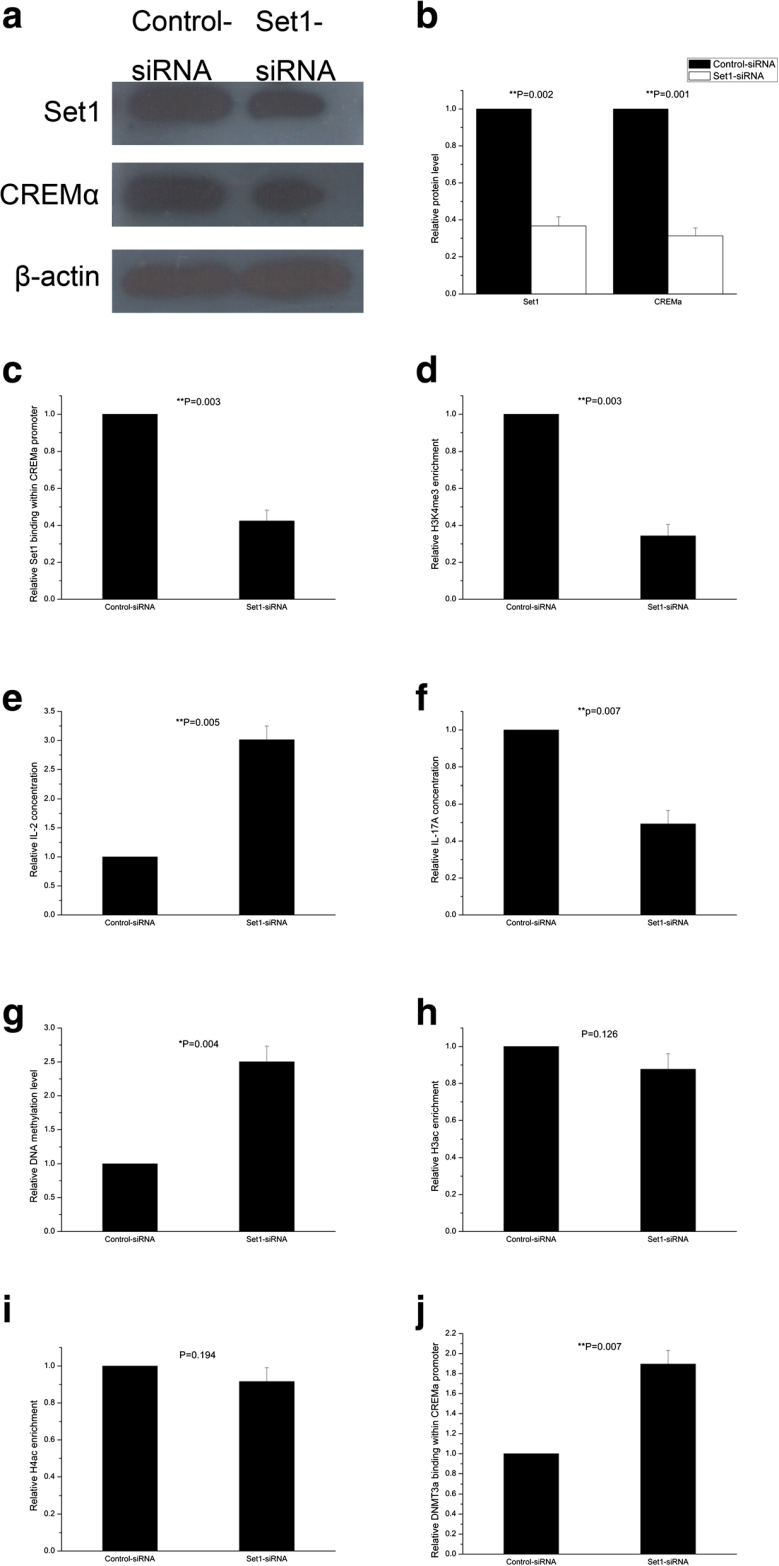
Effects of Set1 down-regulation on CD4^+^ T cells from SLE patients. **a**, **b** Relative Set1 and CREMα protein levels were evaluated by western blotting analysis of SLE CD4^+^ T cells 72 h after transfection with Set1-siRNA or control-siRNA. β-actin served as an endogenous control. **c**, **d** Relative Set1 (**c**) and H3K4me3 (**d**) levels within the CREMα promoter in SLE CD4^+^ T cells transfected with Set1-siRNA or control-siRNA were confirmed by ChIP and real-time PCR 72 h after transfection. Results were normalized to input DNA (total chromatin). **e**, **f** Relative IL-2 (**e**) and IL-17A (**f**) concentrations in the supernatants of SLE CD4^+^ T cells were measured by ELISA 72 h after transfection with Set1-siRNA or control-siRNA. **g** Relative DNA methylation level at the CREMα promoter in SLE CD4^+^ T cells transfected with Set1-siRNA or control-siRNA was assayed by MeDIP and real-time PCR 72 h after transfection. **h**,**i**, **j** Relative enrichments of H3ac (**h**), H4ac (**i**), and DNMT3a (**j**) within the CREMα promoter region in SLE CD4^+^ T cells were tested by ChIP and real-time PCR 72 h after transfection with Set1-siRNA or control-siRNA. Results were normalized to input DNA (total chromatin). All experiments were performed in triplicate

To confirm the effect of Set1 on CREMα expression, we transfected CD4^+^ T cells from three SLE patients with Set1-siRNA or control-siRNA. 72 h after transfection, total amounts of Set1 and CREMα were assessed by Western blotting. As expected, Set1 expression was sharply inhibited by Set1-siRNA compared to the control-siRNA group (Fig. 4a, b), and CREMα level was also down-regulated significantly in CD4^+^ T cells transfected with Set1-siRNA (Fig. 4a, b).

### Down-regulating Set1 in SLE CD4^+^ T cells reduces H3K4me3 enrichment at the promoter of CREMα

In order to ascertain the mechanism whereby Set1 augments CREMα expression, we further analyzed Set1 and H3K4me3 binding at the CREMα promoter in the aforementioned SLE CD4^+^ T cells by ChIP and real-time PCR. After transfection, Set1 binding at the CREMα promoter was also reduced together with total Set1 expression in the Set1-siRNA group (Fig. 4c). Concordantly, H3K4me3 level within the CREMα promoter was decreased after Set1 down-regulation (Fig. 4d).

### Down-regulating Set1 in SLE CD4^+^ T cells induces IL-2 and inhibits IL-17A

Subsequently, we examined the effects of Set1 under-expression on IL-2 and IL-17A productions. 72 h after transfection, supernatant IL-2 and IL-17A concentrations of the SLE CD4^+^ T cells were measured by ELISA. Compared to control-siRNA group, we observed significantly increased IL-2 and deficient IL-17A in the supernatants collected from Set1-siRNA-transfected CD4^+^ T cells (Fig. 4e, f).

### Down-regulating Set1 in SLE CD4^+^ T cells augments DNA methylation at the promoter of CREMα

It is well known that H3K4me3 can suppress DNA methylation and induce histone acetylation [11, 13, 16, 44, 45], so whether the changed H3K4me3 enrichment will alter the levels of DNA methylation and histone acetylation at the CREMα promoter in SLE CD4^+^ T cells is still in question. We measured the quantity of DNA methylation by MeDIP and real-time PCR, and detected the expressions of H3ac and H4ac by ChIP and real-time PCR in the siRNA-transfected SLE CD4^+^ T cells. Compared with the control-siRNA group, DNA methylation at the promoter of CREMα in the Set1-siRNA group was upgraded greatly (Fig. 4g), in addition, H3ac and H4ac enrichments at this region were both mildly decreased, but their changes were not significant (Fig. 4h, i).

### Down-regulating Set1 in SLE CD4^+^ T cells increases DNMT3a binding at the promoter of CREMα

Since the quantity of DNA methylation is increased at the promoter of CREMα in these Set1-siRNA-transfected SLE CD4^+^ T cells, we further assessed DNMT3a binding at the region with ChIP and real-time PCR. Consistent with our finding, the level of DNMT3a was elevated markedly in Set1-siRNA group (Fig. 4j).

### Negatively correlative H3K4me3 enrichment and DNA methylation level at the CREMα promoter in SLE CD4^+^ T cells

**Fig. 5:**
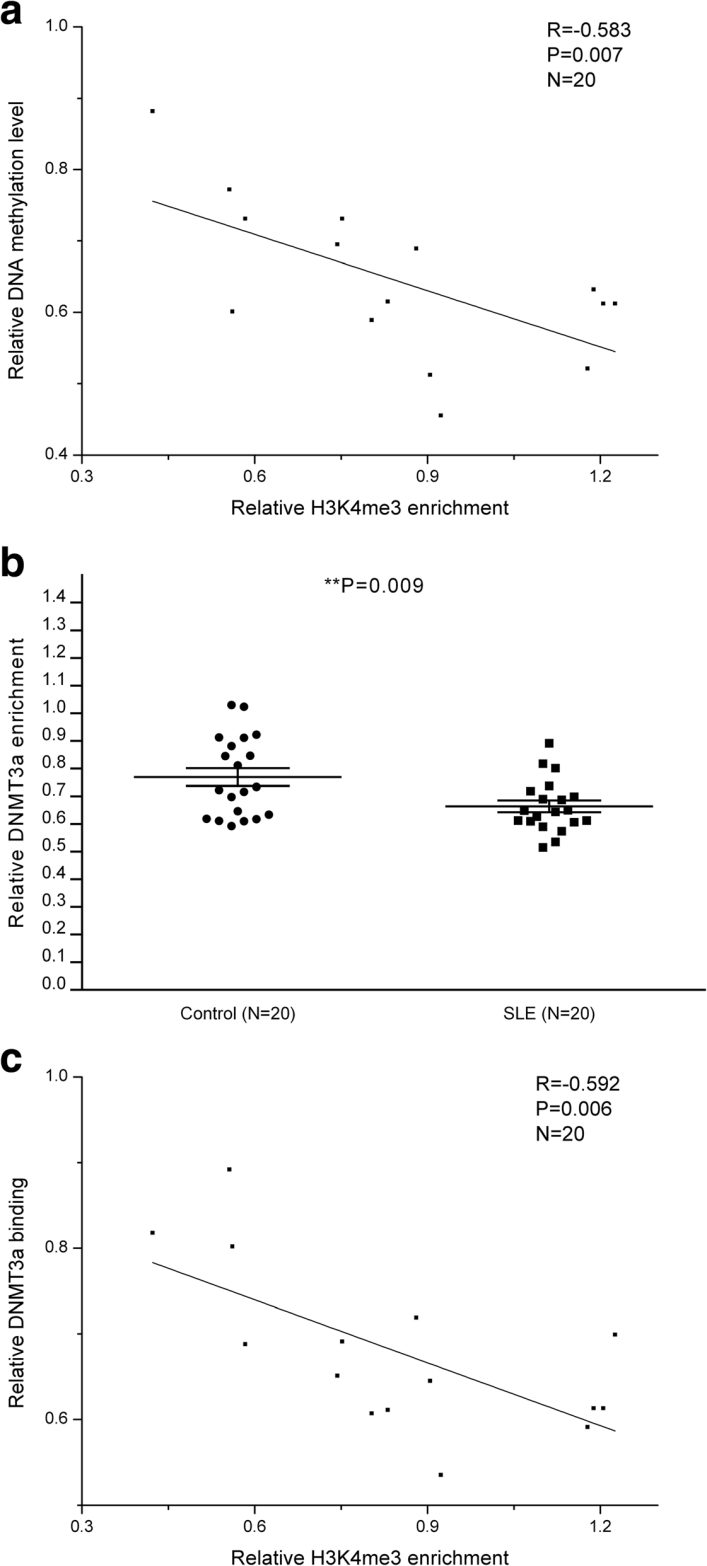
Relationships between H3K4me3, DNA methylation, and DNMT3a. **a** Negative correlation between H3K4me3 enrichment and DNA methylation level at the CREMα promoter in SLE CD4^+^ T cells. **b** Relative level of DNMT3a binding within the CREMα promoter region in CD4^+^ T cells from 20 SLE patients and 20 healthy controls were detected by ChIP and real-time PCR. Results were normalized to input DNA (total chromatin). All data are representative from three independent experiments. **c** Negative correlation between H3K4me3 enrichment and DNMT3a binding at the CREMα promoter in SLE CD4^+^ T cells

Hedrich CM et al. have observed that DNA methylation level at the CREMα promter in SLE CD4^+^ T cells was lower than healthy controls [29]. In order to further investigate the relationship between H3K4me3 and DNA methylation at the CREMα promoter in SLE CD4^+^ T cells, we examined the level of DNA methylation within the CREMα promoter in CD4^+^ T cells from the aforementioned 20 SLE patients via MeDIP and real-time PCR, and proved that H3K4me3 enrichment was negatively correlated with DNA methylation level at the CREMα promoter in SLE CD4^+^ T cells (Fig. 5a, Additional file 1: Table S2).

### Decreased DNMT3a binding at the CREMα promoter in SLE CD4^+^ T cells

We further assayed the expression of DNMT3a within the CREMα promoter in CD4^+^ T cells from the aforementioned 20 SLE patients and 20 healthy controls by ChIP and real-time PCR. Consequently, we unraveled that DNMT3a binding at the CREMα promoter was decreased greatly in SLE CD4^+^ T cells (Fig. 5b, Additional file 1: Table S2), and H3K4me3 enrichment was also negatively correlated with the amount of DNMT3a (Fig. 5c).

## Discussion

In recent years, many researches have focused on the roles of CREMα in the pathogenesis of SLE, especially the mechanisms how CREMα inhibits IL-2 and induces IL-17A. However, the molecular mechanisms causing CREMα increasing in SLE T cells remain elusive.

By ChIP and real-time PCR, we confirmed our ChIP microarray finding that H3K4me3 enrichment at the CREMα promoter in SLE CD4^+^ T cells was significantly higher than in healthy controls. Furthermore, we documented that H3K4me3 enrichment was positively correlated with CREMα mRNA level. These data suggest that elevated H3K4me3 may be the cause of CREMα up-regulation in SLE CD4^+^ T cells. We also proved that Set1 binding at the CREMα promoter was significantly increased in SLE CD4^+^ T cells, and Set1 binding was positively correlated with both H3K4me3 enrichment and CREMα mRNA level. However, there was no difference in MLL1 binding at the CREMα promoter between CD4^+^ T cells from SLE patients and healthy controls. These findings suggest that it is not MLL1, but Set1 overproduction at the CREMα promoter that leads to H3K4me3 up-regulation, which in turn augments the expression of CREMα.

Via siRNA-mediated knocking down, we observed that reducing Set1 in SLE CD4^+^ T cells down-regulated CREMα expression and Set1 binding at the CREMα promoter; accordingly, it decreased H3K4me3 enrichment within the same region, and increased IL-2 concentration, while inhibited IL-17A production. Together, these results indicate that Set1 regulates the expression of CREMα, and this regulation is accomplished at least partly via changing H3K4me3 enrichment at the CREMα promoter; and the up-regulated Set1 binding at the promoter augments the generation of CREMα in SLE CD4^+^ T cells, subsequently results in IL-2 reduction and IL-17A overproduction. Since our manipulations not only altered the amount of Set1 at the CREMα promoter but also affected total Set1 level, we cannot eliminate the possibility that Set1 also regulates CREMα, IL-2, and IL-17A in other ways.

In human, DNA can be methylated by DNMTs (including DNMT1, DNMT3a, and DNMT3b). In this process, DNMTs catalyze the methyl groups to the 5’-carbon position of cytosine residues within CpG dinucleotides, forming 5-methylcytosine bases [8]. H3K4 methylation can down-regulate DNA methylation. It is reported DNMT3a recognizes the unmethylated H3K4 by its ADD domain, subsequently starts de novo DNA methylation [16]. In mutant strains whose H3K4 methylation is diminished, the DNA methylation expression increases fivefold [44]. H3K4me3 also interacts with inhibitor of growth family member 4 (ING4) of histone acetyltransferase binding to ORC-1 (HBO1) [10], Yng1 of NuA3 [46], Esa1 of NuA4 [17, 47], and chromo-ATPase/helicase-DNA binding domain 1 (Chd1) of Spt-Ada-Gcn5 acetyltransferase (SAGA)/SAGA-like (SLIK) [17, 48], thereby recruits these HATs to target genes and enhances their HAT activity. In addition, H3K4me3 can disrupt binding of the nucleosome remodeling and deacetylase (NuRD) to H3 N-terminal tail, consequently preventing target gene deacetylation [49]. It is well-known that DNA methylation can inhibit transcription of gene by changing the chromatin structure to a more compact and inactive form which blocks the access of some transcription factors [50]. On the contrary, histone acetylation can contribute to gene activation through relaxing the structure of chromatin [10, 11].

We have unraveled that H3K4me3 enrichment at the CREMα promoter was elevated in SLE CD4^+^ T cells, therefore we further investigated whether the levels of DNA methylation, DNMT3a, H3ac, and H4ac at this region were affected by the alter of H3K4me3 in these SLE CD4^+^ T cells whose Set1 had been knocked down. We verified that both DNA methylation and DNMT3a at the promoter were up-regulated, while H3ac and H4ac enrichments didn’t change significantly.

Hedrich CM et al. have demonstrated that DNA methylation level at the CREMα promoter in SLE CD4^+^ T cells was down-regulated [29], and our findings are consistent with their result. Taken together, all these data suggested that elevated H3K4me3 at the CREMα promoter excluded DNMT3a, which consequently limited DNA methylation at the same region in SLE CD4^+^ T cells. In order to verify these conclusions, we measured the amounts of DNA methylation and DNMT3a within the CREMα promoter. As our expectation, DNMT3a was down-regulated greatly at the CREMα promoter in SLE CD4^+^ T cells compared to healthy controls; moreover, H3K4me3 enrichment was negatively correlated with both DNA methylation level and DNMT3a binding at the region in SLE CD4^+^ T cells.

## Conclusions

Our results indicate that Set1 binding at the CREMα promoter is upgraded in SLE CD4^+^ T cells, and overexpressed Set1 up-regulates H3K4me3 level within the same region. Elevated H3K4me3 repels DNMT3a, and subsequently inhibited DNA methylation at the domain. All these contribute to CREMα overproduction, and consequently result in IL-2 increasing and IL-17A decreasing, ultimately causing the onset of SLE. Our findings indicate that the epigenetic mechanisms contribute to the development of SLE and provide a novel approach for the treatment of SLE.

## Additional file

Additional file 1: Tables on profiles of SLE patients adopted in ChIP microarray and relevant results of SLE patients. **Table S1.** Profiles of SLE patients adopted in ChIP microarray. **Table S2.** Relevant results of SLE patients. (DOC 61 kb)

## Abbreviations

ACR: American College of Rheumatology
AICD: Activation-induced cell death
cAMP: Cyclic adenosine 5'-monophosphate
Chd1: Chromo-ATPase/helicase-DNA binding domain 1
ChIP: Chromatin immunoprecipitation
COMPASS: Complex of proteins associated with Set1
CREMα: cAMP response element modulator α
DNMT: DNA methyltransferase
FBS: Fetal bovine serum
H3ac: H3 acetylation
H3K4me3: H3 lysine 4 trimethylation
H4ac: H4 acetylation
HAT: Histone acetyltransferase
HBO1: Histone acetyltransferase binding to ORC-1
HCQ: Hydroxychloroquine
HMT: Histone methyltransferase
ING4: Inhibitor of growth family member 4
MeDIP: Methylated CpG-DNA immunoprecipitation
MLL1: Mixed-lineage leukemia 1
NuRD: Nucleosome remodeling and deacetylase
PBMC: Peripheral blood mononuclear cell
Pred: Prednisone
SAGA: Spt-Ada-Gcn5 acetyltransferase
Set1: SET domain containing 1
SLE: Systemic lupus erythematosus
SLEDAI: SLE Disease Activity Index
SLIK: SAGA-like
TG: Tripterygium glycoside
Treg: T regulatory cell
