# Supplementary material for: Increased Set1 binding at the promoter induces aberrant epigenetic alterations and up-regulates cyclic adenosine 5'-monophosphate response element modulator alpha in systemic lupus erythematosus
Source: Clin Epigenetics. 2016 Nov 24;8:126. doi: 10.1186/s13148-016-0294-2 (PMC5122196; doi:10.1186/s13148-016-0294-2)
Supplement: Additional file 1: — Tables on profiles of SLE patients adopted in ChIP microarray and relevant results of SLE patients. Table S1. Profiles of SLE patients adopted in ChIP microarray. Table S2. Relevant results of SLE patients. (DOC 61 kb) [file 13148_2016_294_MOESM1_ESM.doc]

**Table S1** Profiles of SLE patients adopted in ChIP microarray

| Patient | Gender | Age(yrs) | SLEDAI | Medications |
| --- | --- | --- | --- | --- |
| 1 | Female | 23 | 8 | Pred 30mg/d |
| 2 | Female | 20 | 6 | Pred 20mg/d |
| 3 | Male | 38 | 7 | Pred 20mg/d |
| 4 | Female | 27 | 2 | None |
| 5 | Female | 22 | 4 | HCQ 0.2g/d |

SLEDAI: systemic lupus erythematosus; Pred: prednisone; HCQ: hydroxychloroquine

**Table S2** Relevant results of SLE patients

| Patient | CREMα | H3K4me3 | Set1 | MLL1 | DNA methylation | DNMT3a |
| --- | --- | --- | --- | --- | --- | --- |
| 1 | 0.981 | 0.923 | 1.032 | 0.241 | 0.455 | 0.535 |
| 2 | 1.103 | 1.205 | 0.778 | 0.323 | 0.612 | 0.613 |
| 3 | 0.921 | 0.803 | 0.762 | 0.063 | 0.589 | 0.607 |
| 4 | 0.612 | 0.561 | 0.631 | 0.072 | 0.601 | 0.802 |
| 5 | 0.731 | 0.556 | 0.365 | 0.276 | 0.772 | 0.892 |
| 6 | 0.931 | 0.831 | 0.613 | 0.159 | 0.615 | 0.611 |
| 7 | 0.677 | 0.752 | 0.515 | 0.278 | 0.731 | 0.691 |
| 8 | 0.725 | 0.881 | 0.728 | 0.085 | 0.689 | 0.719 |
| 9 | 1.026 | 1.189 | 0.851 | 0.342 | 0.632 | 0.613 |
| 10 | 0.635 | 0.423 | 0.223 | 0.104 | 0.882 | 0.818 |
| 11 | 0.609 | 0.744 | 0.488 | 0.201 | 0.695 | 0.651 |
| 12 | 0.658 | 0.584 | 0.631 | 0.186 | 0.731 | 0.688 |
| 13 | 0.924 | 1.178 | 0.774 | 0.089 | 0.521 | 0.591 |
| 14 | 0.769 | 1.226 | 0.768 | 0.199 | 0.612 | 0.699 |
| 15 | 0.852 | 0.905 | 0.386 | 0.211 | 0.512 | 0.645 |
| 16 | 0.818 | 0.882 | 0.713 | 0.187 | 0.545 | 0.575 |
| 17 | 0.91 | 0.863 | 0.688 | 0.241 | 0.614 | 0.516 |
| 18 | 0.867 | 0.724 | 0.832 | 0.121 | 0.635 | 0.627 |
| 19 | 0.901 | 0.893 | 0.547 | 0.148 | 0.536 | 0.649 |
| 20 | 0.701 | 0.712 | 0.681 | 0.208 | 0.595 | 0.738 |
